# Reducing potential aerosol generation in flexible nasolaryngoscopy: a novel method

**DOI:** 10.1017/S0022215120001413

**Published:** 2020-07-09

**Authors:** J Curran, N Calder, M Yaneza, A Iyer

**Affiliations:** Department of Otolaryngology and Head and Neck Surgery, University Hospital Monklands, Airdrie, Scotland, UK

**Keywords:** Laryngoscopy, Coronavirus, COVID-19, Personal Protective Equipment, Otolaryngologists

## Abstract

**Background:**

Fibre-optic nasoendoscopy and fibre-optic laryngoscopy are high-risk procedures in the coronavirus disease 2019 era, as they are potential aerosol-generating procedures. Barrier protection remains key to preventing transmission.

**Methods:**

A device was developed that patients can wear to reduce potential aerosol contamination of the surroundings.

**Conclusion:**

This device is simple, reproducible, easy to use, economical and well-tolerated. Full personal protection equipment should additionally be worn by the operator.

## Introduction

Fibre-optic nasoendoscopy and fibre-optic laryngoscopy are essential otolaryngology procedures. They allow rapid diagnosis in emergency and elective settings. However, in the coronavirus disease 2019 (Covid-19) era, changes to practice are necessary to protect both the patient and healthcare provider.

Many ENT procedures require personal protective equipment (PPE). Public Health England and ENT UK have published guidance identifying ‘higher risk’ clinical areas. These include areas where nasoendoscopy is performed regularly.^1^ Moreover, ENT clinics do not have laminar airflow rooms; hence, room ventilation is dependent on the frequency of air changes. A standard room has four to six air changes per hour; thus, a room where an aerosol-generating procedure has been performed may be empty for at least 1 hour prior to cleaning, in order to reduce the risk from cross-contamination caused by aerosols.

It is best practice to presume there is a degree of aerosolisation given the concentration of virus particles in the nasal cavity and the potential for ENT procedures to cause coughing or sneezing. Fibre-optic laryngoscopy procedures have the potential to produce aerosols associated with patient coughing, sneezing or talking, or the use of suction. Furthermore, droplets are detectable from the patient in the air when they are speaking, which is often part of the fibre-optic laryngoscopy assessment.^2^

According to current guidance, all persons performing an aerosol-generating procedure must wear PPE, consisting of a filtering face piece code 3 (FFP3) mask, adequate eye protection,^3^ gloves and a fluid-resistant gown.^1^ In the context of an upper airway procedure requiring suction, use of a telemonitor and camera is recommended, instead of the eyepiece on the endoscope, to increase the distance between the operator and patient, and minimise aerosol inhalation. These utilise a barrier strategy to reduce viral transmission to the operator.

Reducing the escape of aerosols from the patient to their surroundings might increase safety and reduce the time lag between the use of clinic rooms. A modified surgical mask was previously described by Workman *et al*. in a study where aerosolisation was simulated in a cadaver.^4^ The findings demonstrated that such masks may reduce droplet production from the patient. We describe a barrier device for patients that is easily made from materials available in hospitals with an anaesthetic or emergency department; this device is cost-effective, easy to use and well-tolerated by the patient.

## Technical description

**Fig. 1.**
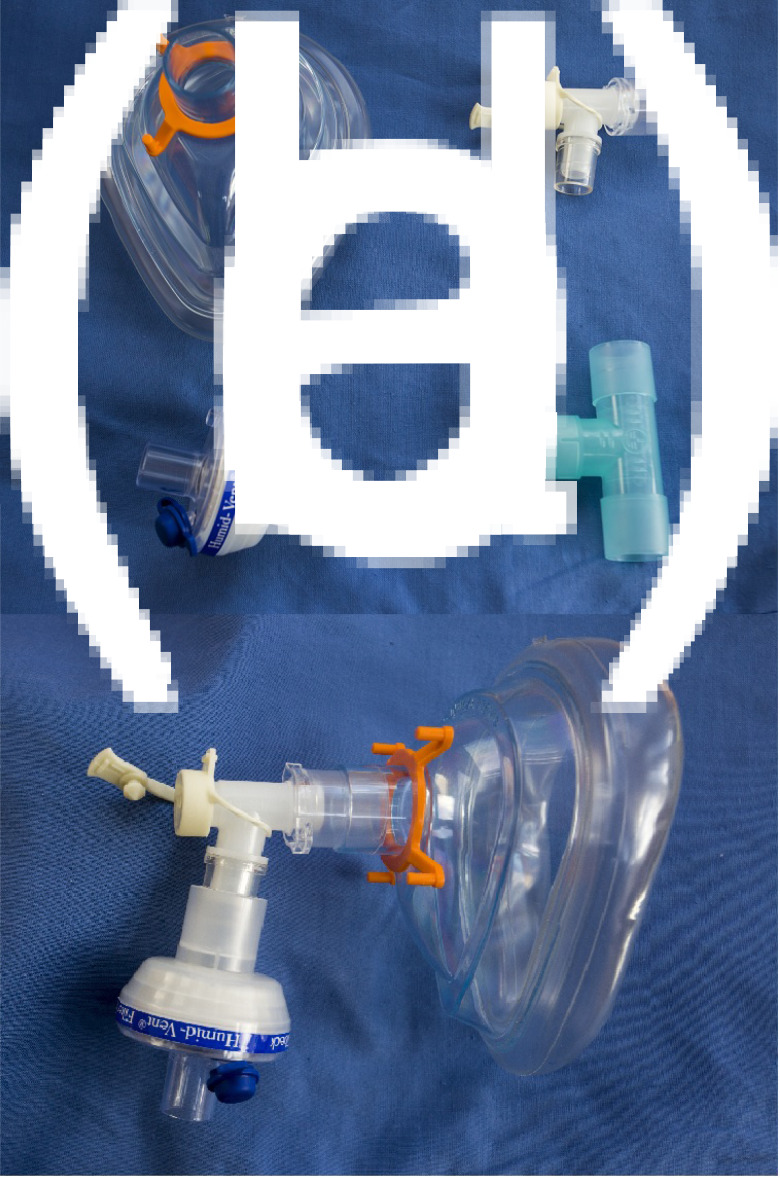
Device assembly. A = mask; B = DAR connector; C = filter; D = T-piece; E = assembled device

**Fig. 2.**
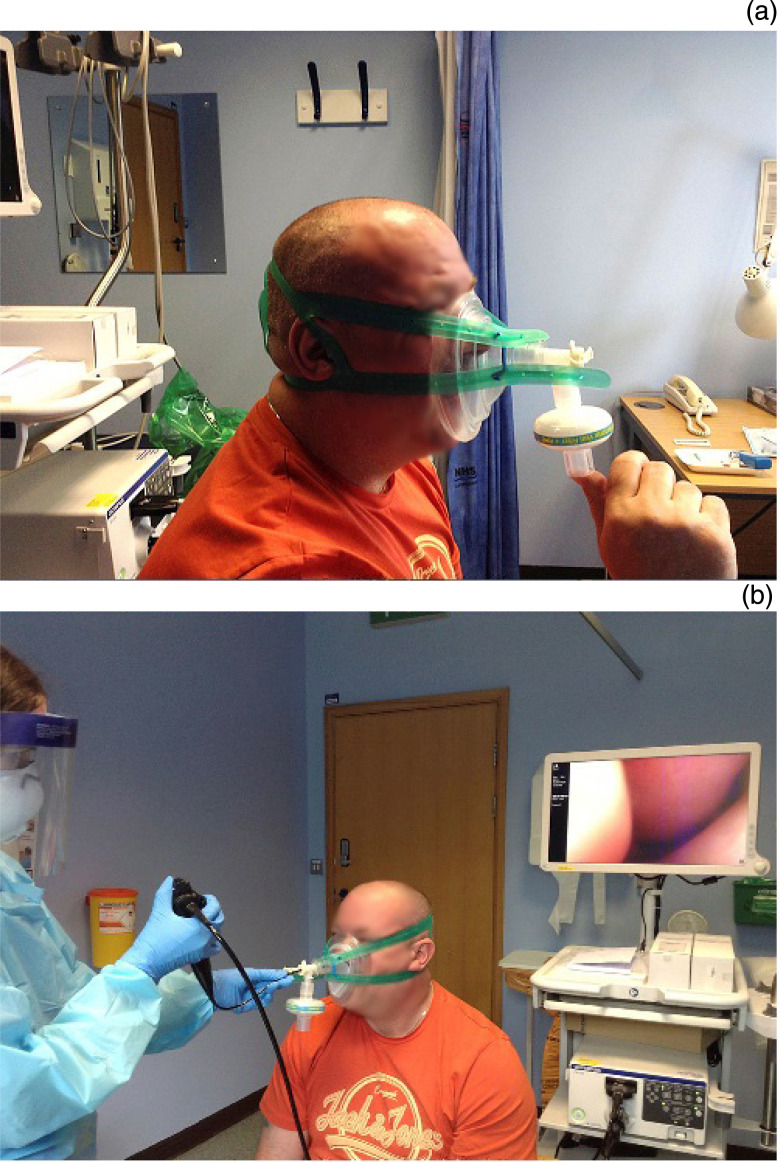
Photographs showing: (a) the patient checking the seal, and (b) the device in use during nasolaryngoscopy. Published with patient's permission.

The device is composed of an anaesthetic ‘closed’ facemask, anaesthetic filter, DAR^™^ (Covidien) connector (or similar), which is L-shaped with a closable hole for instruments (alternatively, a T-piece 22 mm connector can be used), and a reusable harness attachment for continuous positive airway pressure (CPAP) (Vygon, Swindon, UK) (Figure 1). The local cost for all disposable pieces (which excludes the CPAP harness) totals £1.86. The filter, and the DAR connector and T-piece, are attached to the closed facemask as seen in Figure 1, with the filter facing inferiorly. If using a T-piece, a seal is made with the ‘finger’ of a glove attached, and a small hole is made with a thick needle for passing the endoscope. The mask is inverted to align the hole with the nostrils instead of the mouth, and it is secured in place with the CPAP harness (Figure 2).

The ‘closed’ anaesthetic masks come in many sizes. Sizes 3–4 are generally suitable for women and sizes 5–6 for men. The correct size is best ascertained by measuring the distance between the nasal bridge and a point below the lower lip where it is fully covered by the sides of the mask. For edentulous patients, dentures are left in place. Applying a thick coat of gel lubrication to patients with facial hair can encourage a seal for these patients.

The device was tested for comfort by the authors and a barrier seal was verified by manually covering the anaesthetic filter. This can be replicated with every patient to test whether there is an appropriate fit (Figure 2a). It should be impossible for the patient to breath in when the filter is covered. Nasoendoscopy is performed as demonstrated in Figure 2b.

Full PPE as described above should still be used, as well as a camera and monitor where able. Local anaesthetic applied on a cotton patty (not spray) should be used before attaching the mask, in order to reduce sneezing or coughing. The scope could be withdrawn whilst holding a cleaning wipe to reduce the chance of droplets falling and contaminating the air.

After the procedure, the scope is removed and sent for cleaning as per local protocols. The mask and attachments should be discarded, apart from the harness, which can be reused after cleaning with appropriate wipes for 60 seconds (or as per local policy).

## Discussion

This device provides a means to help to reduce viral transmission and provides ENT staff with additional protection. It likely reduces the chance of aerosol spread and contamination of the clinic room. It is simple, reproducible, and easy to make with easily available resources. Unlike previously described masks,^4^ this mask does not use valuable PPE, it provides an air-tight seal and does not run the risk of potential injury to the patient associated with the use of staples.

A survey was conducted by the unit using a questionnaire. This comprised 10 questions with Likert scores between 1 and 5, with 5 reflecting great agreement and 1 representing no agreement at all. The questionnaire was completed by 10 patients and the surgeons (Table 1).

**Table 1.** Questions and mean scores for flexible nasolaryngoscopy using anaesthetic face mask assembly

Questions	Mean score
Patient's questions	
– I am satisfied with the outcome of endoscopy	5
– The procedure was comfortable	4.4
– Wearing the mask itself was not causing any major problems	4.4
– The time taken for the procedure was satisfactory	4.8
– I would have the procedure again if needed	4.9
– I would recommend this procedure to a friend	5
Surgeon's questions	
– I am satisfied with the outcome of endoscopy	4.5
– The time taken for the procedure was satisfactory	4.2
– The procedure was comfortable to perform with the mask	4.4
– I would do the procedure again if needed using this technique	4.8

For each question, patients and surgeons were asked ‘to what extent do you agree with the following statement?’, with responses being: 5 = strongly agree, 4 = agree, 3 = no opinion, 2 = disagree and 1 = strongly disagree.

The questionnaire findings revealed a score of 4.4 for comfort and 5 for overall satisfaction. Surgeons using the mask gave a score of 4.5 for overall satisfaction and 4.2 for the time taken to perform the procedure. All other aspects of the procedure also scored very well. Of note, only once was the procedure abandoned on a patient using this mask, because of extremely narrow nostrils; however, this patient found the mask comfortable.

Some limitations of this paper are that the device may be uncomfortable for some patients; nevertheless, it has been well-tolerated by the authors and patients in the clinic to date, as evidenced by our survey. Some hospitals may not have operating theatre or emergency departments, and thus, they may need to order some of the equipment required. These items should be on ordering lists as they are all standard pieces on emergency trolleys, apart from the CPAP harness. Furthermore, assembly of the device and verification of the seal will lengthen the procedure time and may not be appropriate in some emergency settings. Lastly, humidification in a closed circuit may present an issue where demisting agents are not available. The authors suggest gently pressing the lens of the endoscope on a clear area of the inferior turbinate, to clear large debris or eliminate misting.

Areas for future research include an assessment of the impact that using this device has on clinic times. A pre-made supply of the most common mask sizes could be created to minimise delay in clinics. Further research could examine the device's practicality in emergency settings. More importantly, one could test whether wearing this mask prevents aerosolisation completely.
